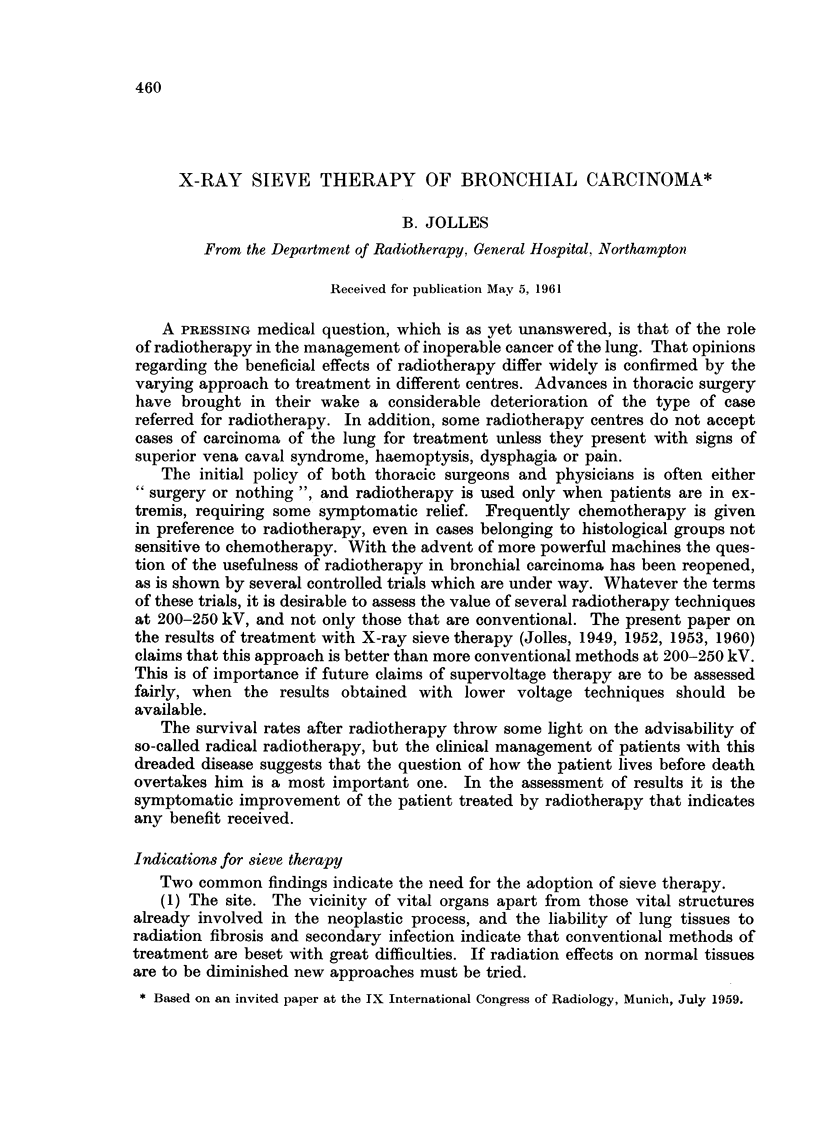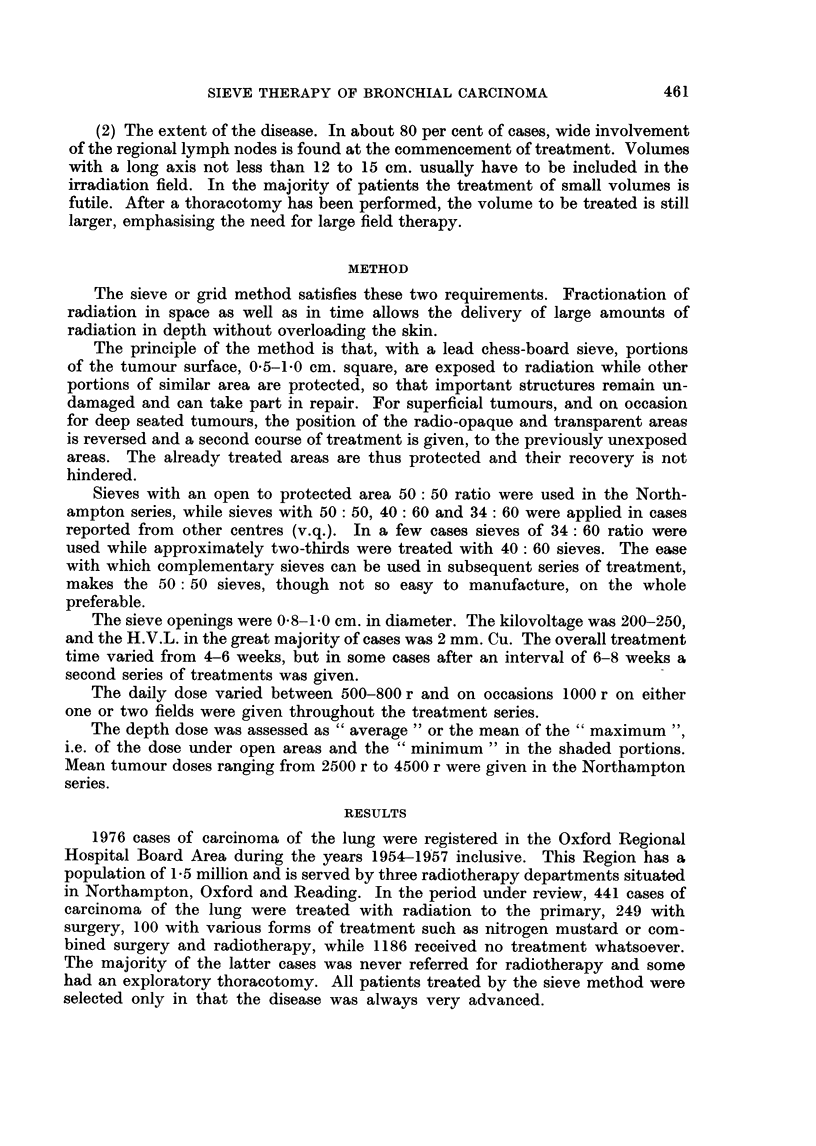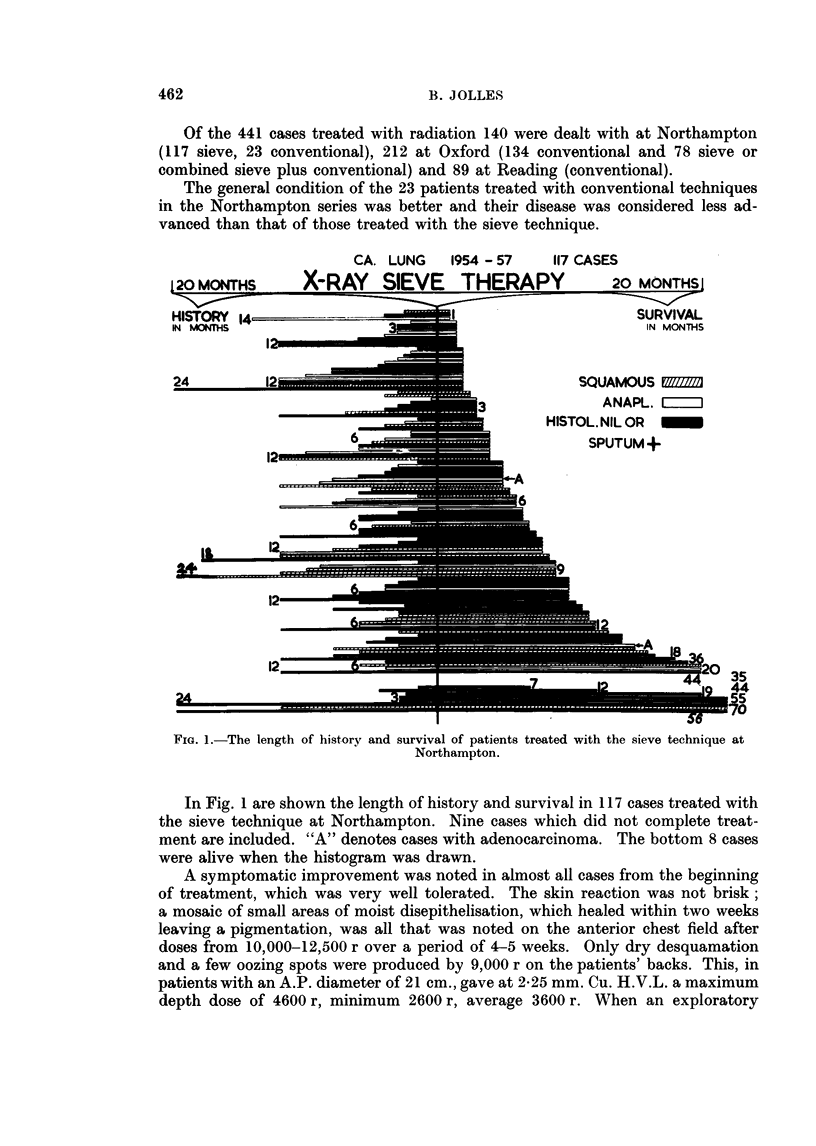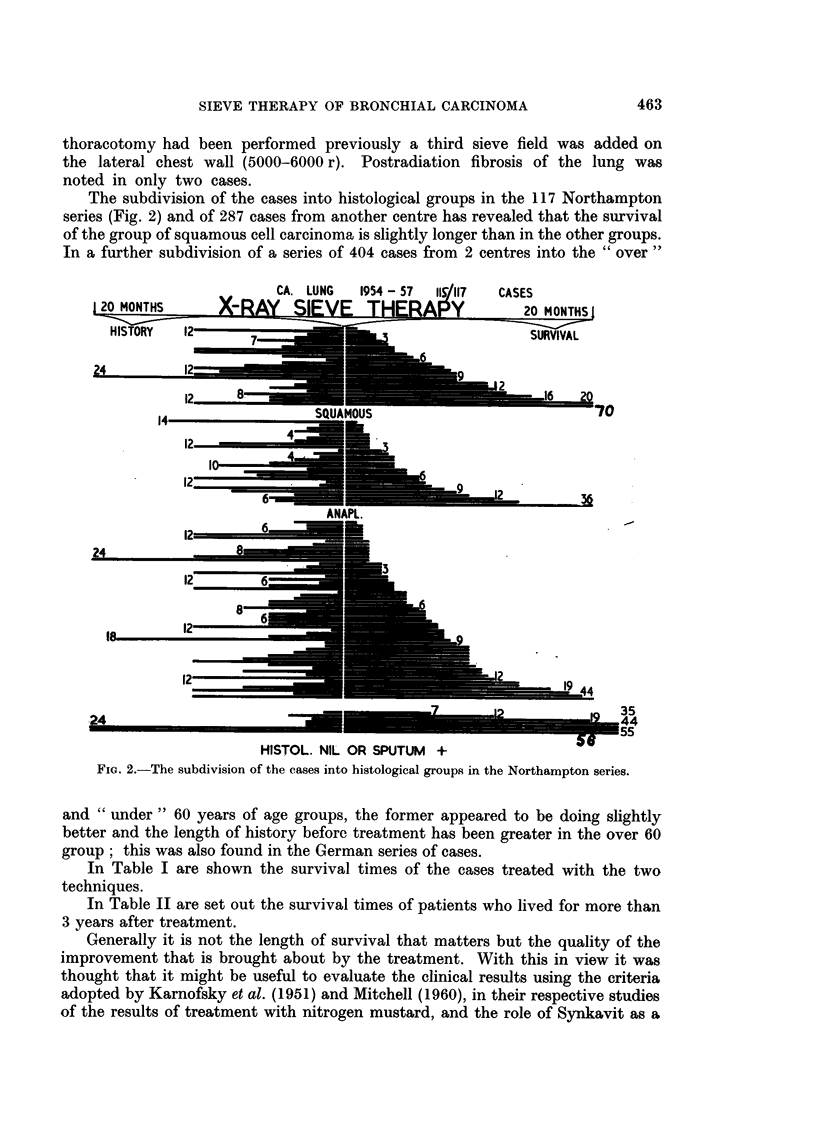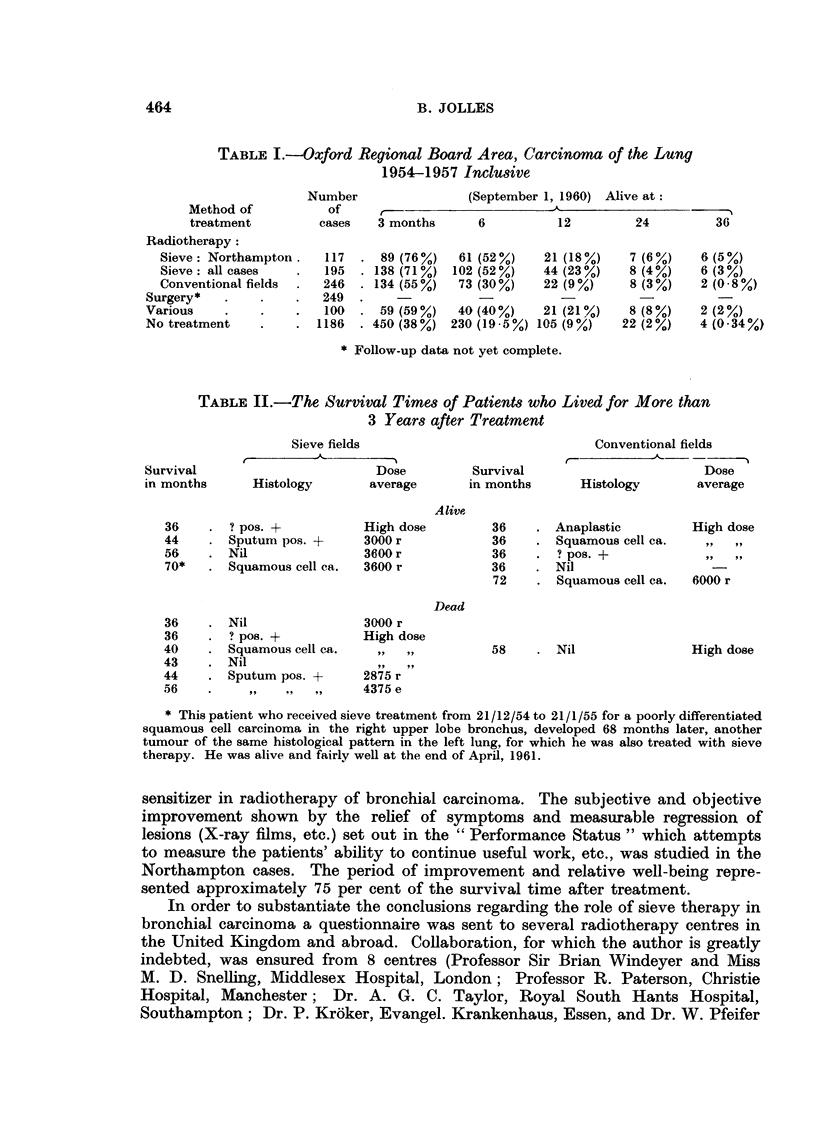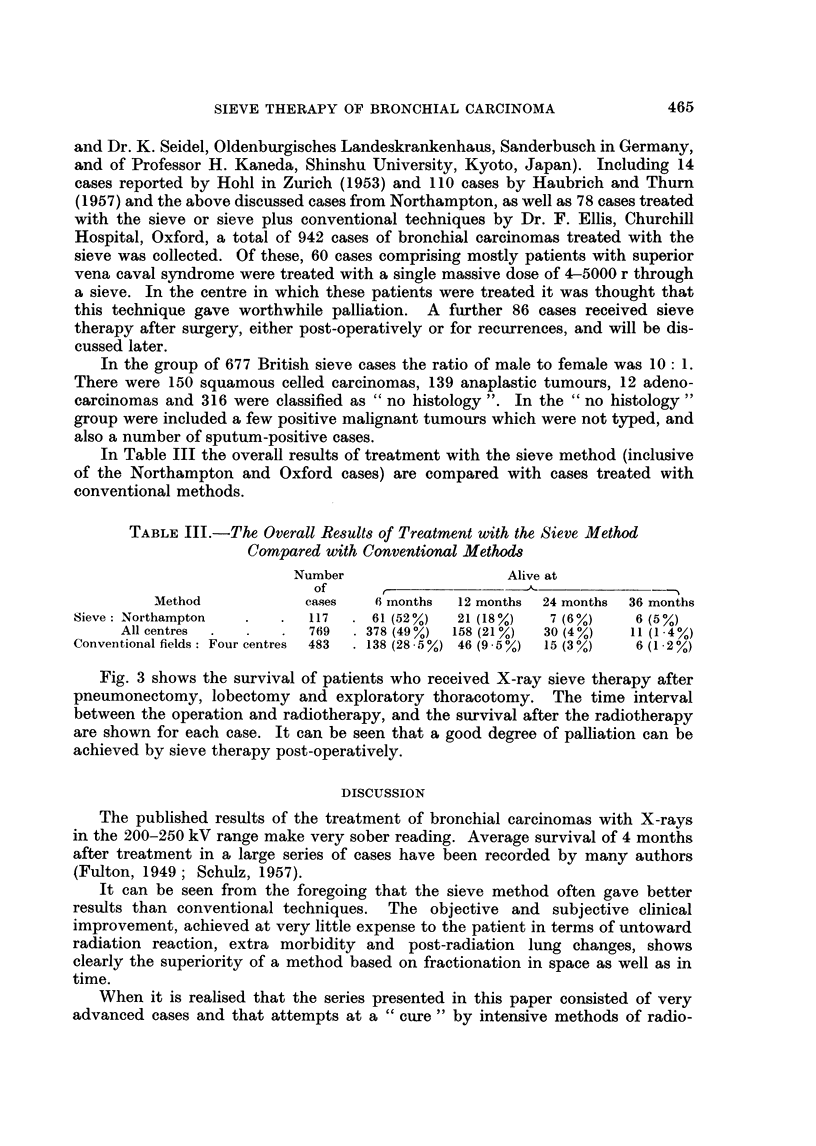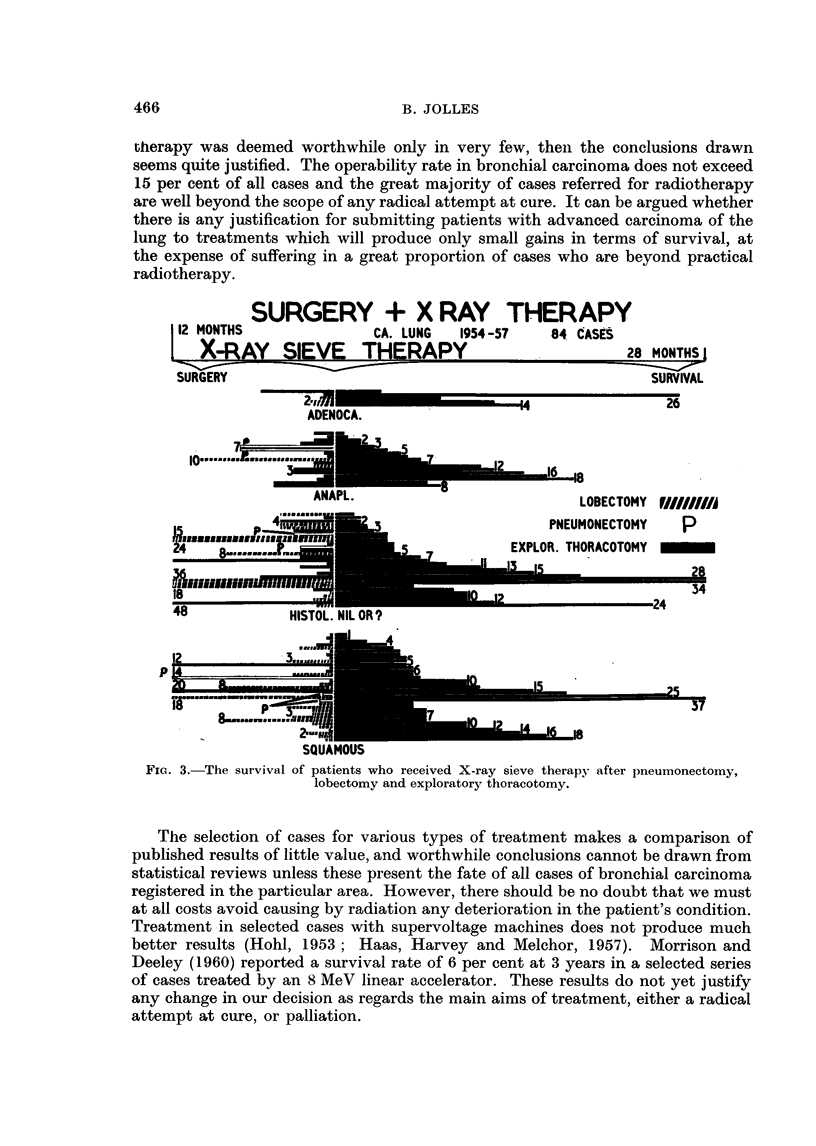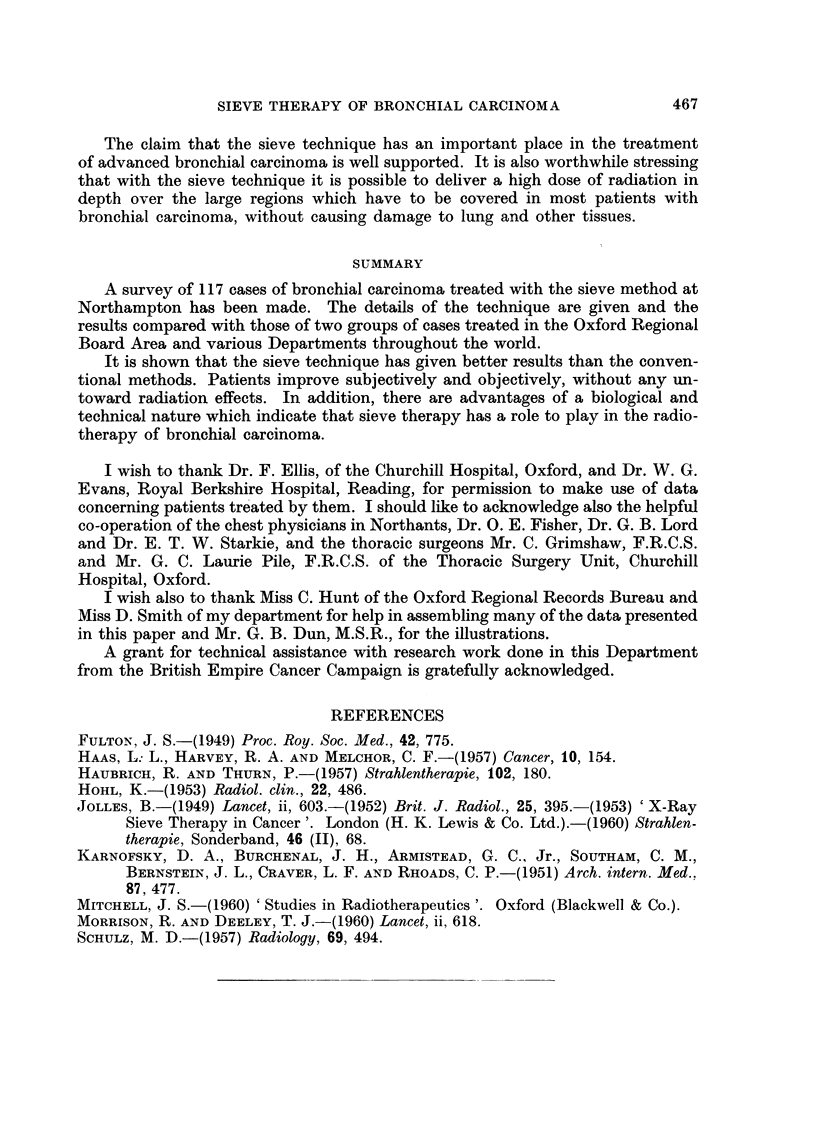# X-Ray Sieve Therapy of Bronchial Carcinoma*

**DOI:** 10.1038/bjc.1961.55

**Published:** 1961-09

**Authors:** B. Jolles


### X-RAY SIEVE THERAPY OF BRONCHIAL CARCINOMA*

B. JOLLES

*From the Department of Radiotherapy, General Hospital, Northampton*

Received for publication May 5, 1961

A PRESSING medical question, which is as yet unanswered, is that of the role of radiotherapy in the management of inoperable cancer of the lung. That opinions regarding the beneficial effects of radiotherapy differ widely is confirmed by the varying approach to treatment in different centres. Advances in thoracic surgery have brought in their wake a considerable deterioration of the type of case referred for radiotherapy. In addition, some radiotherapy centres do not accept cases of carcinoma of the lung for treatment unless they present with signs of superior vena caval syndrome, haemoptysis, dysphagia or pain.

The initial policy of both thoracic surgeons and physicians is often either " surgery or nothing ", and radiotherapy is used only when patients are in extremis, requiring some symptomatic relief. Frequently chemotherapy is given in preference to radiotherapy, even in cases belonging to histological groups not sensitive to chemotherapy. With the advent of more powerful machines the question of the usefulness of radiotherapy in bronchial carcinoma has been reopened, as is shown by several controlled trials which are under way. Whatever the terms of these trials, it is desirable to assess the value of several radiotherapy techniques at 200–250 kV, and not only those that are conventional. The present paper on the results of treatment with X-ray sieve therapy (Jolles, 1949, 1952, 1953, 1960) claims that this approach is better than more conventional methods at 200–250 kV. This is of importance if future claims of supervoltage therapy are to be assessed fairly, when the results obtained with lower voltage techniques should be available.

The survival rates after radiotherapy throw some light on the advisability of so-called radical radiotherapy, but the clinical management of patients with this dreaded disease suggests that the question of how the patient lives before death overtakes him is a most important one. In the assessment of results it is the symptomatic improvement of the patient treated by radiotherapy that indicates any benefit received.

### *Indications for sieve therapy*

Two common findings indicate the need for the adoption of sieve therapy.

(1) The site. The vicinity of vital organs apart from those vital structures already involved in the neoplastic process, and the liability of lung tissues to radiation fibrosis and secondary infection indicate that conventional methods of treatment are beset with great difficulties. If radiation effects on normal tissues are to be diminished new approaches must be tried.

* Based on an invited paper at the IX International Congress of Radiology, Munich, July 1959.

(2) The extent of the disease. In about 80 per cent of cases, wide involvement of the regional lymph nodes is found at the commencement of treatment. Volumes with a long axis not less than 12 to 15 cm. usually have to be included in the irradiation field. In the majority of patients the treatment of small volumes is futile. After a thoracotomy has been performed, the volume to be treated is still larger, emphasising the need for large field therapy.

### METHOD

The sieve or grid method satisfies these two requirements. Fractionation of radiation in space as well as in time allows the delivery of large amounts of radiation in depth without overloading the skin.

The principle of the method is that, with a lead chess-board sieve, portions of the tumour surface, 0·5–1·0 cm. square, are exposed to radiation while other portions of similar area are protected, so that important structures remain undamaged and can take part in repair. For superficial tumours, and on occasion for deep seated tumours, the position of the radio-opaque and transparent areas is reversed and a second course of treatment is given, to the previously unexposed areas. The already treated areas are thus protected and their recovery is not hindered.

Sieves with an open to protected area 50 : 50 ratio were used in the Northampton series, while sieves with 50 : 50, 40 : 60 and 34 : 60 were applied in cases reported from other centres (v.q.). In a few cases sieves of 34 : 60 ratio were used while approximately two-thirds were treated with 40 : 60 sieves. The ease with which complementary sieves can be used in subsequent series of treatment, makes the 50 : 50 sieves, though not so easy to manufacture, on the whole preferable.

The sieve openings were 0·8–1·0 cm. in diameter. The kilovoltage was 200–250, and the H.V.L. in the great majority of cases was 2 mm. Cu. The overall treatment time varied from 4–6 weeks, but in some cases after an interval of 6–8 weeks a second series of treatments was given.

The daily dose varied between 500–800 r and on occasions 1000 r on either one or two fields were given throughout the treatment series.

The depth dose was assessed as " average " or the mean of the " maximum ", i.e. of the dose under open areas and the " minimum " in the shaded portions. Mean tumour doses ranging from 2500 r to 4500 r were given in the Northampton series.

### RESULTS

1976 cases of carcinoma of the lung were registered in the Oxford Regional Hospital Board Area during the years 1954–1957 inclusive. This Region has a population of 1·5 million and is served by three radiotherapy departments situated in Northampton, Oxford and Reading. In the period under review, 441 cases of carcinoma of the lung were treated with radiation to the primary, 249 with surgery, 100 with various forms of treatment such as nitrogen mustard or combined surgery and radiotherapy, while 1186 received no treatment whatsoever. The majority of the latter cases was never referred for radiotherapy and some had an exploratory thoracotomy. All patients treated by the sieve method were selected only in that the disease was always very advanced.

Of the 441 cases treated with radiation 140 were dealt with at Northampton (117 sieve, 23 conventional), 212 at Oxford (134 conventional and 78 sieve or combined sieve plus conventional) and 89 at Reading (conventional).

The general condition of the 23 patients treated with conventional techniques in the Northampton series was better and their disease was considered less advanced than that of those treated with the sieve technique.

FIG. 1.—The length of history and survival of patients treated with the sieve technique at Northampton.

In Fig. 1 are shown the length of history and survival in 117 cases treated with the sieve technique at Northampton. Nine cases which did not complete treatment are included. "A" denotes cases with adenocarcinoma. The bottom 8 cases were alive when the histogram was drawn.

A symptomatic improvement was noted in almost all cases from the beginning of treatment, which was very well tolerated. The skin reaction was not brisk ; a mosaic of small areas of moist disepithelisation, which healed within two weeks leaving a pigmentation, was all that was noted on the anterior chest field after doses from 10,000–12,500 r over a period of 4–5 weeks. Only dry desquamation and a few oozing spots were produced by 9,000 r on the patients' backs. This, in patients with an A.P. diameter of 21 cm., gave at 2·25 mm. Cu. H.V.L. a maximum depth dose of 4600 r, minimum 2600 r, average 3600 r. When an exploratory thoracotomy had been performed previously a third sieve field was added on the lateral chest wall (5000–6000 r). Postradiation fibrosis of the lung was noted in only two cases.

The subdivision of the cases into histological groups in the 117 Northampton series (Fig. 2) and of 287 cases from another centre has revealed that the survival of the group of squamous cell carcinoma is slightly longer than in the other groups. In a further subdivision of a series of 404 cases from 2 centres into the " over "

FIG. 2.—The subdivision of the cases into histological groups in the Northampton series.

and " under " 60 years of age groups, the former appeared to be doing slightly better and the length of history before treatment has been greater in the over 60 group ; this was also found in the German series of cases.

In Table I are shown the survival times of the cases treated with the two techniques.

In Table II are set out the survival times of patients who lived for more than 3 years after treatment.

Generally it is not the length of survival that matters but the quality of the improvement that is brought about by the treatment. With this in view it was thought that it might be useful to evaluate the clinical results using the criteria adopted by Karnofsky *et al.* (1951) and Mitchell (1960), in their respective studies of the results of treatment with nitrogen mustard, and the role of Synkavit as a

TABLE I.—*Oxford Regional Board Area, Carcinoma of the Lung 1954–1957 Inclusive*

| Method of treatment | Number of cases | (September 1, 1960) Alive at : 3 months | 6 | 12 | 24 | 36 |
|---|---|---|---|---|---|---|
| Radiotherapy : | | | | | | |
| Sieve : Northampton | 117 | 89 (76%) | 61 (52%) | 21 (18%) | 7 (6%) | 6 (5%) |
| Sieve : all cases | 195 | 138 (71%) | 102 (52%) | 44 (23%) | 8 (4%) | 6 (3%) |
| Conventional fields | 246 | 134 (55%) | 73 (30%) | 22 (9%) | 8 (3%) | 2 (0·8%) |
| Surgery* | 249 | — | — | — | — | — |
| Various | 100 | 59 (59%) | 40 (40%) | 21 (21%) | 8 (8%) | 2 (2%) |
| No treatment | 1186 | 450 (38%) | 230 (19·5%) | 105 (9%) | 22 (2%) | 4 (0·34%) |

* Follow-up data not yet complete.

TABLE II.—*The Survival Times of Patients who Lived for More than 3 Years after Treatment*

| Sieve fields: Survival in months | Histology | Dose average | Conventional fields: Survival in months | Histology | Dose average |
|---|---|---|---|---|---|
| *Alive* | | | | | |
| 36 | ? pos. + | High dose | 36 | Anaplastic | High dose |
| 44 | Sputum pos. + | 3000 r | 36 | Squamous cell ca. | „ „ |
| 56 | Nil | 3600 r | 36 | ? pos. + | „ „ |
| 70* | Squamous cell ca. | 3600 r | 36 | Nil | — |
| | | | 72 | Squamous cell ca. | 6000 r |
| *Dead* | | | | | |
| 36 | Nil | 3000 r | | | |
| 36 | ? pos. + | High dose | | | |
| 40 | Squamous cell ca. | „ „ | 58 | Nil | High dose |
| 43 | Nil | „ „ | | | |
| 44 | Sputum pos. + | 2875 r | | | |
| 56 | „ „ „ | 4375 e | | | |

* This patient who received sieve treatment from 21/12/54 to 21/1/55 for a poorly differentiated squamous cell carcinoma in the right upper lobe bronchus, developed 68 months later, another tumour of the same histological pattern in the left lung, for which he was also treated with sieve therapy. He was alive and fairly well at the end of April, 1961.

sensitizer in radiotherapy of bronchial carcinoma. The subjective and objective improvement shown by the relief of symptoms and measurable regression of lesions (X-ray films, etc.) set out in the " Performance Status " which attempts to measure the patients' ability to continue useful work, etc., was studied in the Northampton cases. The period of improvement and relative well-being represented approximately 75 per cent of the survival time after treatment.

In order to substantiate the conclusions regarding the role of sieve therapy in bronchial carcinoma a questionnaire was sent to several radiotherapy centres in the United Kingdom and abroad. Collaboration, for which the author is greatly indebted, was ensured from 8 centres (Professor Sir Brian Windeyer and Miss M. D. Snelling, Middlesex Hospital, London ; Professor R. Paterson, Christie Hospital, Manchester ; Dr. A. G. C. Taylor, Royal South Hants Hospital, Southampton ; Dr. P. Kröker, Evangel. Krankenhaus, Essen, and Dr. W. Pfeifer and Dr. K. Seidel, Oldenburgisches Landeskrankenhaus, Sanderbusch in Germany, and of Professor H. Kaneda, Shinshu University, Kyoto, Japan). Including 14 cases reported by Hohl in Zurich (1953) and 110 cases by Haubrich and Thurn (1957) and the above discussed cases from Northampton, as well as 78 cases treated with the sieve or sieve plus conventional techniques by Dr. F. Ellis, Churchill Hospital, Oxford, a total of 942 cases of bronchial carcinomas treated with the sieve was collected. Of these, 60 cases comprising mostly patients with superior vena caval syndrome were treated with a single massive dose of 4–5000 r through a sieve. In the centre in which these patients were treated it was thought that this technique gave worthwhile palliation. A further 86 cases received sieve therapy after surgery, either post-operatively or for recurrences, and will be discussed later.

In the group of 677 British sieve cases the ratio of male to female was 10 : 1. There were 150 squamous celled carcinomas, 139 anaplastic tumours, 12 adenocarcinomas and 316 were classified as " no histology ". In the " no histology " group were included a few positive malignant tumours which were not typed, and also a number of sputum-positive cases.

In Table III the overall results of treatment with the sieve method (inclusive of the Northampton and Oxford cases) are compared with cases treated with conventional methods.

TABLE III.—*The Overall Results of Treatment with the Sieve Method Compared with Conventional Methods*

| Method | Number of cases | Alive at 6 months | 12 months | 24 months | 36 months |
|---|---|---|---|---|---|
| Sieve : Northampton | 117 | 61 (52%) | 21 (18%) | 7 (6%) | 6 (5%) |
| All centres | 769 | 378 (49%) | 158 (21%) | 30 (4%) | 11 (1·4%) |
| Conventional fields : Four centres | 483 | 138 (28·5%) | 46 (9·5%) | 15 (3%) | 6 (1·2%) |

Fig. 3 shows the survival of patients who received X-ray sieve therapy after pneumonectomy, lobectomy and exploratory thoracotomy. The time interval between the operation and radiotherapy, and the survival after the radiotherapy are shown for each case. It can be seen that a good degree of palliation can be achieved by sieve therapy post-operatively.

### DISCUSSION

The published results of the treatment of bronchial carcinomas with X-rays in the 200–250 kV range make very sober reading. Average survival of 4 months after treatment in a large series of cases have been recorded by many authors (Fulton, 1949 ; Schulz, 1957).

It can be seen from the foregoing that the sieve method often gave better results than conventional techniques. The objective and subjective clinical improvement, achieved at very little expense to the patient in terms of untoward radiation reaction, extra morbidity and post-radiation lung changes, shows clearly the superiority of a method based on fractionation in space as well as in time.

When it is realised that the series presented in this paper consisted of very advanced cases and that attempts at a " cure " by intensive methods of radiotherapy was deemed worthwhile only in very few, then the conclusions drawn seems quite justified. The operability rate in bronchial carcinoma does not exceed 15 per cent of all cases and the great majority of cases referred for radiotherapy are well beyond the scope of any radical attempt at cure. It can be argued whether there is any justification for submitting patients with advanced carcinoma of the lung to treatments which will produce only small gains in terms of survival, at the expense of suffering in a great proportion of cases who are beyond practical radiotherapy.

FIG. 3.—The survival of patients who received X-ray sieve therapy after pneumonectomy, lobectomy and exploratory thoracotomy.

The selection of cases for various types of treatment makes a comparison of published results of little value, and worthwhile conclusions cannot be drawn from statistical reviews unless these present the fate of all cases of bronchial carcinoma registered in the particular area. However, there should be no doubt that we must at all costs avoid causing by radiation any deterioration in the patient's condition. Treatment in selected cases with supervoltage machines does not produce much better results (Hohl, 1953 ; Haas, Harvey and Melchor, 1957). Morrison and Deeley (1960) reported a survival rate of 6 per cent at 3 years in a selected series of cases treated by an 8 MeV linear accelerator. These results do not yet justify any change in our decision as regards the main aims of treatment, either a radical attempt at cure, or palliation.

The claim that the sieve technique has an important place in the treatment of advanced bronchial carcinoma is well supported. It is also worthwhile stressing that with the sieve technique it is possible to deliver a high dose of radiation in depth over the large regions which have to be covered in most patients with bronchial carcinoma, without causing damage to lung and other tissues.

### SUMMARY

A survey of 117 cases of bronchial carcinoma treated with the sieve method at Northampton has been made. The details of the technique are given and the results compared with those of two groups of cases treated in the Oxford Regional Board Area and various Departments throughout the world.

It is shown that the sieve technique has given better results than the conventional methods. Patients improve subjectively and objectively, without any untoward radiation effects. In addition, there are advantages of a biological and technical nature which indicate that sieve therapy has a role to play in the radiotherapy of bronchial carcinoma.

I wish to thank Dr. F. Ellis, of the Churchill Hospital, Oxford, and Dr. W. G. Evans, Royal Berkshire Hospital, Reading, for permission to make use of data concerning patients treated by them. I should like to acknowledge also the helpful co-operation of the chest physicians in Northants, Dr. O. E. Fisher, Dr. G. B. Lord and Dr. E. T. W. Starkie, and the thoracic surgeons Mr. C. Grimshaw, F.R.C.S. and Mr. G. C. Laurie Pile, F.R.C.S. of the Thoracic Surgery Unit, Churchill Hospital, Oxford.

I wish also to thank Miss C. Hunt of the Oxford Regional Records Bureau and Miss D. Smith of my department for help in assembling many of the data presented in this paper and Mr. G. B. Dun, M.S.R., for the illustrations.

A grant for technical assistance with research work done in this Department from the British Empire Cancer Campaign is gratefully acknowledged.

### REFERENCES

FULTON, J. S.—(1949) *Proc. Roy. Soc. Med.*, **42**, 775.

HAAS, L. L., HARVEY, R. A. AND MELCHOR, C. F.—(1957) *Cancer*, **10**, 154.

HAUBRICH, R. AND THURN, P.—(1957) *Strahlentherapie*, **102**, 180.

HOHL, K.—(1953) *Radiol. clin.*, **22**, 486.

JOLLES, B.—(1949) *Lancet*, ii, 603.—(1952) *Brit. J. Radiol.*, **25**, 395.—(1953) ' X-Ray Sieve Therapy in Cancer '. London (H. K. Lewis & Co. Ltd.).—(1960) *Strahlentherapie*, Sonderband, **46** (II), 68.

KARNOFSKY, D. A., BURCHENAL, J. H., ARMISTEAD, G. C., Jr., SOUTHAM, C. M., BERNSTEIN, J. L., CRAVER, L. F. AND RHOADS, C. P.—(1951) *Arch. intern. Med.*, **87**, 477.

MITCHELL, J. S.—(1960) ' Studies in Radiotherapeutics '. Oxford (Blackwell & Co.).

MORRISON, R. AND DEELEY, T. J.—(1960) *Lancet*, ii, 618.

SCHULZ, M. D.—(1957) *Radiology*, **69**, 494.